# Pulmonary hypertension secondary to IgG4-related disease: a case report and literature review

**DOI:** 10.1093/omcr/omag151

**Published:** 2026-11-02

**Authors:** Mohammed Alkhanafsa, Jamil Wafi, Osayd Mosleh, Mohammed Alkhatib, Shanima Ismail, Miral Hamed

**Affiliations:** Department of Internal Medicine, Hamad Medical Corporation, Hamad Medical City, Al Rayyan Road, Al Sadd, P.O. Box 3050, Doha, Qatar; Department of Internal Medicine, Hamad Medical Corporation, Hamad Medical City, Al Rayyan Road, Al Sadd, P.O. Box 3050, Doha, Qatar; Department of Internal Medicine, Hamad Medical Corporation, Hamad Medical City, Al Rayyan Road, Al Sadd, P.O. Box 3050, Doha, Qatar; Department of Pulmonary Medicine, Hamad Medical Corporation, Hamad Medical City, Al Rayyan Road, Al Sadd, P.O. Box 3050, Doha, Qatar; Department of Pulmonary Medicine, Hamad Medical Corporation, Hamad Medical City, Al Rayyan Road, Al Sadd, P.O. Box 3050, Doha, Qatar; Department of Rheumatology, Hamad Medical Corporation, Hamad Medical City, Al Rayyan Road, Al Sadd, P.O. Box 3050, Doha, Qatar

**Keywords:** IgG4-related disease, pulmonary hypertension, rituximab, pre-capillary pulmonary hypertension, case report

## Abstract

IgG4-related disease (IgG4-RD) is a fibroinflammatory condition that can involve multiple organs, including the kidneys, but pulmonary vascular involvement is rare. We present a 29-year-old Nepalese male with known IgG4-RD kidney involvement who developed progressive exertional dyspnea after two years of clinical stability on azathioprine. Chest imaging showed no underlying interstitial lung disease. Evaluation revealed severe pre-capillary pulmonary hypertension (PH) on right heart catheterization, with negative workup for common PH etiologies. After limited response to corticosteroid and azathioprine intensification, rituximab induction, followed by six-monthly maintenance cycles, was given while sildenafil and macitentan were continued. This led to marked clinical, biochemical, and hemodynamic improvement: NT-proBNP decreased from 1333 to 24 pg/mL, RVSP improved from 68 to 42.61 mmHg, and IgG4 decreased from 10.732 to 1.186 g/L. This case highlights that IgG4-RD can cause severe pre-capillary PH and may respond substantially to rituximab.

## Introduction

IgG4-related disease (IgG4-RD) is a systemic immune-mediated condition characterized by tumefactive lesions, lymphoplasmacytic infiltration rich in IgG4-positive plasma cells, storiform fibrosis, and often elevated serum IgG4 levels [1, 2]. While the pancreas, salivary glands, and kidneys are commonly involved, pulmonary manifestations typically present as interstitial lung disease or airway nodules [3]. Pulmonary hypertension (PH) secondary to IgG4-RD is exceedingly rare. We report a case of severe pre-capillary PH in a patient with established IgG4-RD kidney involvement that responded dramatically to rituximab. The absence of interstitial lung disease on chest imaging was important because interstitial lung disease is a recognized pulmonary manifestation of IgG4-RD and may independently contribute to PH.

## Case presentation

A 29-year-old Nepalese male with known IgG4-RD kidney involvement was maintained on azathioprine 100 mg daily and remained clinically stable for two years. The original renal biopsy showed plasma cell-rich chronic tubulointerstitial inflammation with interstitial fibrosis, storiform areas, tubular injury, and focal tubular atrophy. Immunohistochemistry showed increased IgG4-positive plasma cells (>20/high-power field), with an IgG4/IgG-positive plasma-cell ratio > 40%. He then developed progressive exertional dyspnea. He was a lifelong non-smoker with no prior pulmonary disease. He denied dry cough, leg swelling, syncope, chest pain, orthopnea, fever, or weight loss, and physical examination showed no elevated jugular venous pressure or peripheral edema.

Electrocardiography showed right bundle branch block, right axis deviation, and biatrial enlargement. N-terminal pro-B-type natriuretic peptide (NT-proBNP) was elevated at 1333 pg/mL. Transthoracic echocardiography (TTE) revealed severe right atrial and right ventricular dilatation with preserved systolic function, with tricuspid annular plane systolic excursion (TAPSE) of 2.1 cm. Estimated right ventricular systolic pressure (RVSP) was 68 mmHg, with tricuspid regurgitation velocity (TRV) of 4.04 m/s, and preserved left ventricular ejection fraction (LVEF) of 55%. Chest computed tomography (CT) showed dilated right-sided cardiac chambers and enlarged pulmonary arteries, consistent with PH. No interstitial lung disease, pulmonary fibrosis, honeycombing, or significant parenchymal infiltrative lung disease was seen on chest imaging.

Immunoglobulin testing revealed elevated total IgG (35 g/L), markedly increased IgG3 (>8500 mg/L), and elevated IgG4 (10.732 g/L). Baseline laboratory and diagnostic investigations are summarized in Table 1. The patient was receiving azathioprine 100 mg daily; prednisolone 40 mg daily was started for 2 weeks, followed by tapering by 5 mg weekly for a total duration of 9 weeks. Azathioprine was increased to 150 mg daily. Positron emission tomography-computed tomography (PET-CT) demonstrated mild to moderate hypermetabolic activity in slightly enlarged lymph nodes above and below the diaphragm (Fig. 1). A detailed chronological treatment and response timeline is provided in Table 2.

**Table 1:** Laboratory and diagnostic investigations with reference ranges.

Investigation	Result	Reference range/normal finding
NT-proBNP	1333 pg/mL at baseline; 24 pg/mL after treatment	<125 pg/mL
Total IgG	35 g/L at baseline; 13.41 g/L after treatment	7–16 g/L
IgG3	>8500 mg/L at baseline; 704 mg/L after treatment	210–1760 mg/L
IgG4	10.732 g/L at baseline; 1.186 g/L after treatment	0.03–2.01 g/L
Antinuclear antibody (ANA)	Negative	Negative
Anti-neutrophil cytoplasmic antibody (ANCA)	Negative	Negative
Anti-double-stranded DNA (anti-dsDNA)	Negative	Negative
Anti-cyclic citrullinated peptide antibody (anti-CCP)	Negative	Negative
Antiphospholipid antibodies	Negative	Negative
Human immunodeficiency virus (HIV) testing	Negative	Negative

**Figure 1:**
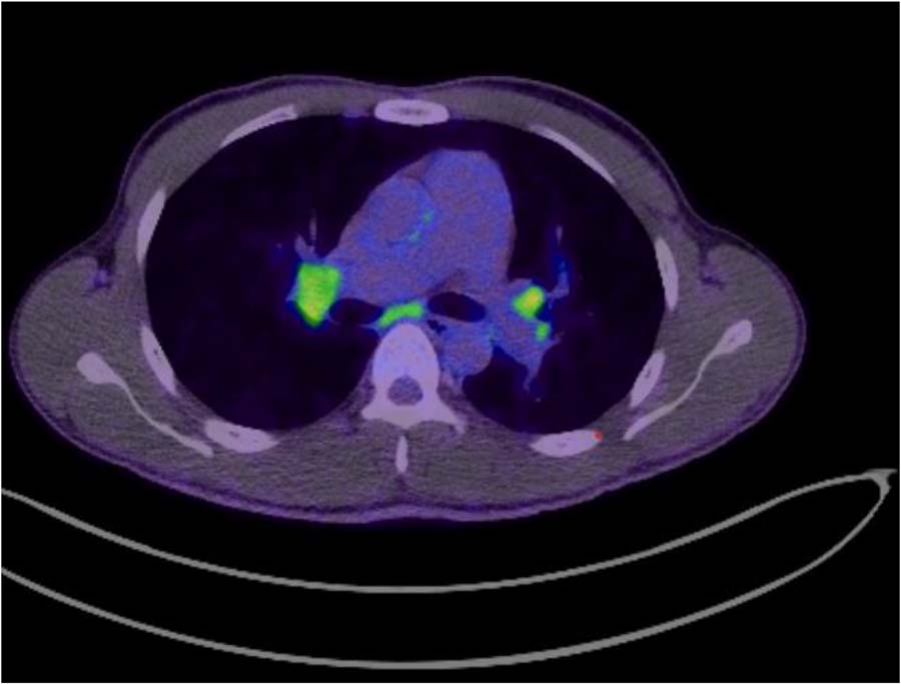
Baseline PET-CT demonstrates hypermetabolic lymph nodes above and below the diaphragm, consistent with active systemic IgG4-RD.

**Table 2:** Chronological treatment timeline and clinical, hemodynamic, laboratory, and imaging response.

Relative time point	Treatment/intervention	Key findings	Symptoms/clinical status
Week 0: Baseline presentation	The patient was receiving azathioprine 100 mg daily. Prednisolone 40 mg daily was started for 2 weeks, followed by tapering by 5 mg weekly for a total duration of 9 weeks. Azathioprine was increased to 150 mg daily.	TTE: severe right atrial and right ventricular dilatation, preserved RV systolic function (TAPSE 2.1 cm), RVSP 68 mmHg, and LVEF 55%. RHC/mPAP: not yet performed. Labs: NT-proBNP 1333 pg/mL, total IgG 35 g/L, IgG3 > 8500 mg/L, and IgG4 10.732 g/L. PET-CT: hypermetabolic lymph nodes above and below the diaphragm, consistent with active systemic disease.	Progressive exertional dyspnea only; no dry cough, leg swelling, elevated jugular venous pressure, or peripheral edema.
Week 4: During corticosteroid therapy	RHC was performed while the patient was receiving corticosteroid therapy. Severe pre-capillary PH was confirmed, and sildenafil plus macitentan were initiated.	RHC: mPAP 48 mmHg, PCWP 7 mmHg, PVR 15 Wood units, CO 2.5 L/min, and CI 1.65 L/min/m^2^. There was no significant vasoreactivity to inhaled nitric oxide.	Persistent symptomatic PH.
Week 9: Completion of corticosteroid taper	The patient completed the corticosteroid taper and continued sildenafil plus macitentan. Because of persistent radiological disease activity, rituximab induction was planned.	Chest CT: no significant interval radiological improvement and no underlying interstitial lung disease. TTE/RVSP: not performed at this time. RHC/mPAP: not repeated.	Ongoing disease activity despite corticosteroid therapy and pulmonary vasodilator treatment.
Weeks 10–12: Rituximab induction	Rituximab induction was administered as two intravenous doses of 1 g, given 2 weeks apart. Sildenafil and macitentan were continued.	TTE: partial improvement, with RVSP decreasing from 68 to 52 mmHg. RHC/mPAP: not repeated.	Improved exercise tolerance.
Six months after rituximab induction	Rituximab was repeated as maintenance therapy. Azathioprine, low-dose prednisolone, sildenafil, and macitentan were continued.	TTE: RVSP decreased further from 68 to 42.61 mmHg, with normalization of right ventricular function. RHC/mPAP: not repeated. Labs: NT-proBNP decreased from 1333 to 24 pg/mL, total IgG from 35 to 13.41 g/L, IgG3 from > 8500 to 704 mg/L, and IgG4 from 10.732 to 1.186 g/L. PET-CT: resolution of previously hypermetabolic lymph nodes.	Marked clinical improvement with sustained functional recovery and no new cardiopulmonary symptoms.

Autoimmune screening, including antinuclear antibody (ANA), anti-neutrophil cytoplasmic antibody (ANCA), anti-double-stranded DNA (anti-dsDNA), anti-cyclic citrullinated peptide antibody (anti-CCP), and antiphospholipid antibodies, was negative. Ventilation/perfusion (V/Q) scan excluded chronic thromboembolic PH. Human immunodeficiency virus (HIV) testing, liver disease evaluation, and pulmonary function tests (PFTs) were unremarkable. Bronchoscopy with bronchoalveolar lavage (BAL) showed a mixed inflammatory pattern with mild eosinophilia (55% macrophages, 25% lymphocytes, 12% neutrophils, and 8% eosinophils), with no infection or malignancy.

Right heart catheterization (RHC), performed during corticosteroid therapy, confirmed severe pre-capillary PH, as evidenced by mean pulmonary artery pressure (mPAP) of 48 mmHg, pulmonary capillary wedge pressure (PCWP) of 7 mmHg, pulmonary vascular resistance (PVR) of 15 Wood units, cardiac output (CO) of 2.5 L/min, and cardiac index (CI) of 1.65 L/min/m^2^. There was no significant vasoreactivity to inhaled nitric oxide. After comprehensive exclusion of alternative causes, including connective tissue disease, chronic thromboembolic disease, left heart disease, HIV infection, liver disease, and interstitial lung disease, the findings were deemed most consistent with PH secondary to underlying IgG4-RD. The patient was started on targeted PH therapy with sildenafil and macitentan. There was no clear major symptomatic improvement before rituximab; exertional dyspnea and symptomatic PH persisted despite corticosteroid therapy and initiation of pulmonary vasodilator therapy.

After completion of the corticosteroid taper at Week 9, repeat chest CT showed no significant radiological improvement, and no echocardiogram was performed at that time. Given persistent radiological disease activity despite corticosteroid therapy, azathioprine intensification, and continued sildenafil plus macitentan, rituximab was initiated as induction therapy with two intravenous doses of 1 g administered two weeks apart during Weeks 10–12. During follow-up after rituximab induction, while sildenafil and macitentan were continued, the patient reported improved exercise tolerance. Follow-up TTE showed partial improvement, with RVSP decreasing from 68 to 52 mmHg. He was subsequently maintained on prednisolone 5 mg daily and azathioprine 150 mg daily, with a repeat rituximab cycle scheduled at six months.

Six months after rituximab induction, rituximab was repeated as maintenance therapy. The patient demonstrated marked clinical, biochemical, and radiological improvement. NT-proBNP decreased from 1333 to 24 pg/mL. Repeat TTE showed improvement in pulmonary pressures, with RVSP reduced from 68 to 42.61 mmHg, along with normalization of right ventricular function. Immunoglobulin profiling showed a substantial response: total IgG decreased from 35 to 13.41 g/L, IgG3 from > 8500 to 704 mg/L, and IgG4 from 10.732 to 1.186 g/L. Repeat PET-CT demonstrated interval resolution of previously identified hypermetabolic lymph nodes above and below the diaphragm, in keeping with radiological remission of active disease (Fig. 2).

**Figure 2:**
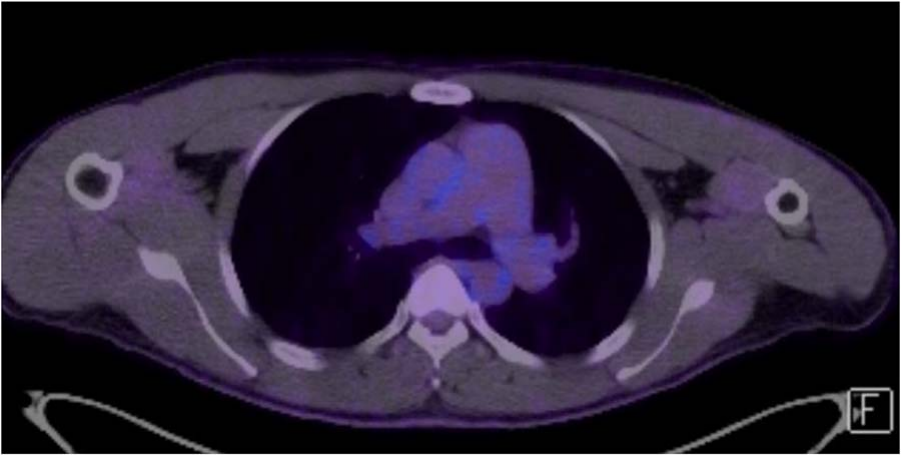
Follow-up PET-CT after rituximab demonstrates resolution of previously hypermetabolic lymph nodes, consistent with radiological remission.

The patient continues on maintenance azathioprine (150 mg daily), low-dose prednisolone (2.5 mg daily), sildenafil, and macitentan, with planned six-monthly rituximab cycles. He remains clinically stable with sustained functional improvement and no new cardiopulmonary symptoms during follow-up.

## Discussion

Our patient had established IgG4-RD kidney involvement maintained on azathioprine for two years when he developed progressive exertional dyspnea. Comprehensive evaluation excluded chronic thromboembolic disease (normal V/Q scan), connective tissue disease (negative ANA, ANCA, anti-CCP, anti-dsDNA, and antiphospholipid antibodies), left heart disease (normal PCWP of 7 mmHg), HIV infection, liver disease, and interstitial lung disease. RHC confirmed severe pre-capillary PH (mPAP 48 mmHg, PVR 15 Wood units, CI 1.65 L/min/m^2^) with no significant vasoreactivity to inhaled nitric oxide. The hemodynamic pattern and negative vasoreactivity argued against idiopathic pulmonary arterial hypertension.

The absence of interstitial lung disease is clinically important. IgG4-RD lung involvement more commonly manifests as interstitial lung disease, inflammatory nodules, bronchovascular thickening, or airway disease rather than isolated pulmonary vascular disease [3]. In this patient, chest CT showed no interstitial lung disease or fibrotic parenchymal lung disease, reducing the likelihood that PH was driven by hypoxic or parenchymal lung mechanisms.

The pathophysiology of PH in IgG4-RD is not fully understood but several mechanisms have been proposed [4]. First, direct inflammatory infiltration of pulmonary arterial walls by IgG4-positive plasma cells and lymphocytes can lead to luminal narrowing and increased vascular resistance. Second, cytokine-mediated vascular remodeling, particularly involving interleukin-6 (IL-6) and transforming growth factor-beta (TGF-beta), both implicated in inflammatory fibrosis, may promote smooth muscle proliferation and endothelial dysfunction. Third, extrinsic vascular compression by fibrotic or lymphadenopathic mediastinal disease may occur. In our patient, PET-CT demonstrated hypermetabolic lymphadenopathy above and below the diaphragm but no discrete mediastinal mass compressing the pulmonary arteries, favoring a vasculopathic or cytokine-driven mechanism rather than extrinsic compression.

The published literature on IgG4-RD-associated PH remains very limited. A directly comparable report by Yamamoto et al. described PH associated with IgG4-RD, supporting the concept that IgG4-RD can rarely involve the pulmonary vasculature [4]. To provide a broader comparison, we also reviewed selected thoracic IgG4-RD literature. Large clinicopathologic and radiologic series have described IgG4-RD lung and pleural involvement, including nodular, interstitial, bronchovascular, and pleural patterns [3, 8, 9]. These reports are important because they show that parenchymal lung disease is a recognized thoracic manifestation of IgG4-RD; however, our patient had no interstitial lung disease, pulmonary fibrosis, or major parenchymal lung disease on chest imaging. This makes the present case different from typical IgG4-RD lung disease and supports a predominantly pulmonary vascular phenotype. Overall, the current case is notable for invasive hemodynamic confirmation of severe pre-capillary PH, exclusion of common PH etiologies, absence of interstitial lung disease, limited response to corticosteroid and azathioprine intensification, and longitudinal improvement after rituximab induction and maintenance while sildenafil and macitentan were continued. Table 3 summarizes the comparison with selected published thoracic IgG4-RD literature.

**Table 3:** Comparison with selected published thoracic IgG4-RD literature relevant to pulmonary hypertension.

Report	Clinical context	Relevance to PH or thoracic disease	Comparison with current case
**Yamamoto et al.** [4]	Reported PH associated with IgG4-RD.	Supports pulmonary vascular involvement as a rare manifestation of IgG4-RD.	Most directly comparable to the present case because PH was linked to IgG4-RD. Our case adds detailed RHC data, serial RVSP/NT-proBNP follow-up, absence of ILD, and response after rituximab-based treatment.
**Matsui et al.** [3]	Review of IgG4-RD lung disease.	Summarized common thoracic phenotypes such as ILD, nodules, and airway/bronchovascular involvement.	Relevant because it highlights that ILD may occur in IgG4-RD. In contrast, our patient had no ILD, making parenchymal lung disease unlikely as the cause of PH.
**Zen et al.** [8]	Clinicopathologic series of IgG4-RD lung and pleural disease.	Described lung and pleural involvement within the IgG4-RD spectrum.	Useful comparison for thoracic IgG4-RD patterns. Our case differs because pulmonary vascular disease predominated without significant parenchymal or pleural disease.
**Inoue et al.** [9]	Radiologic-pathologic series describing CT patterns of IgG4-RD lung disease.	Reported CT patterns including nodules, ground-glass change, interstitial change, and bronchovascular bundle involvement.	Supports the importance of assessing CT for ILD or infiltrative lung disease. Our chest imaging did not show these findings, strengthening the pulmonary vascular interpretation.
Current case	Established IgG4-RD kidney involvement with severe pre-capillary PH and no ILD on chest CT.	RHC showed mPAP 48 mmHg, PCWP 7 mmHg, PVR 15 Wood units, and CI 1.65 L/min/m^2^; workup for alternative PH etiologies was negative.	Adds a clear chronological treatment-response profile: limited improvement with corticosteroid/azathioprine and PH therapy alone, followed by symptomatic, biochemical, echocardiographic, and PET-CT improvement after rituximab while sildenafil and macitentan were continued.

First-line immunosuppression for IgG4-RD includes glucocorticoids, with steroid-sparing agents such as azathioprine, mycophenolate mofetil, or methotrexate used for maintenance [5]. However, our patient had persistent radiological activity despite prednisolone 40 mg daily with taper and an increased azathioprine dose of 150 mg daily. This lack of adequate response prompted consideration of rituximab, a B-cell-depleting anti-CD20 monoclonal antibody. Rituximab has emerged as highly effective in refractory IgG4-RD, with a landmark prospective open-label study demonstrating response in 97% of patients and complete remission in 83% at six months [6]. The rationale for rituximab use is based on the central role of B cells and plasmablasts in IgG4-RD pathogenesis; rituximab eliminates CD20-positive B cells, thereby reducing autoantibody production, antigen presentation, and cytokine secretion [7].

The therapeutic response in this case should be interpreted in the context of combined treatment. Sildenafil and macitentan were initiated after RHC and could have contributed to reduced pulmonary vascular resistance, improved right ventricular loading, and lower RVSP. However, the most pronounced clinical and biomarker improvement occurred after rituximab induction and maintenance, alongside normalization of right ventricular function, NT-proBNP reduction from 1333 to 24 pg/mL, IgG4 reduction from 10.732 to 1.186 g/L, and PET-CT resolution of hypermetabolic lymphadenopathy. This parallel improvement in cardiopulmonary, immunological, and radiological parameters supports rituximab-mediated control of the underlying inflammatory IgG4-RD process as a major contributor to recovery, while acknowledging the additive role of pulmonary vasodilator therapy.

This case adds to the very limited literature on IgG4-RD-associated PH and provides important clinical lessons. First, PH can develop even in patients with established IgG4-RD on conventional immunosuppression. Second, absence of interstitial lung disease does not exclude IgG4-RD-associated PH, because pulmonary vascular mechanisms may predominate. Third, failure of corticosteroids and azathioprine does not indicate treatment resistance to all agents; rituximab can be highly effective. Fourth, six-monthly maintenance rituximab may sustain remission and prevent relapse. Fifth, PH in this context may be partially reversible when systemic inflammation is adequately controlled, although pulmonary vasodilator therapy may also contribute to hemodynamic improvement. Close multidisciplinary follow-up involving rheumatology, cardiology, and pulmonology is essential for monitoring disease activity, pulmonary pressures, and treatment-related complications.

## Conclusion

Pulmonary hypertension is a rare but severe complication of IgG4-RD that can occur even in patients on conventional immunosuppression. This case demonstrates that pre-capillary PH secondary to IgG4-RD may be refractory to corticosteroids and azathioprine but can show a robust and sustained response after B-cell depletion with rituximab, with normalization of cardiac biomarkers, improved pulmonary pressures, and systemic remission. The improvement should be interpreted in the setting of combined therapy, as sildenafil and macitentan may also have contributed to hemodynamic improvement. Maintenance rituximab every six months may sustain this response.

## Key clinical message

In patients with IgG4-RD who develop unexplained dyspnea, PH should be suspected and investigated with echocardiography and RHC. The absence of interstitial lung disease on chest imaging does not exclude IgG4-RD-associated PH. When conventional immunosuppression fails, rituximab induction followed by six-monthly maintenance therapy may achieve both immunological and hemodynamic improvement, particularly when used in multidisciplinary care alongside appropriate PH-directed therapy.

## Data Availability

All data supporting the findings of this case report are included within the manuscript. Additional data may be available from the corresponding author upon reasonable request, subject to patient confidentiality and institutional regulations.
